# Comparative analysis of chloroplast genomes of 29 tomato germplasms: genome structures, phylogenetic relationships, and adaptive evolution

**DOI:** 10.3389/fpls.2023.1179009

**Published:** 2023-05-09

**Authors:** Xiaomin Wang, Shengyi Bai, Zhaolei Zhang, Fushun Zheng, Lina Song, Lu Wen, Meng Guo, Guoxin Cheng, Wenkong Yao, Yanming Gao, Jianshe Li

**Affiliations:** ^1^ College of Enology and Horticultrue, Ningxia University, Yinchuan, China; ^2^ Ningxia Modern Facility Horticulture Engineering Technology Research Center, Ningxia Facility Horticulture (Ningxia University) Technology Innovation Center, Yinchuan, China; ^3^ Key Laboratory of Modern Molecular Breeding for Dominant and Special Crops in Ningxia, Ningxia University, Yinchuan, China; ^4^ Hebei Key Laboratory of Study and Exploitation of Chinese Medicine, Chengde Medical University, Chengde, China

**Keywords:** tomato, germplasm, chloroplast genome, phylogenetic, adaptive evolution

## Abstract

In order to compare and analyze the chloroplast (cp) genomes of tomato germplasms and understand their phylogenetic relationships, the cp genomes of 29 tomato germplasms were sequenced and analyzed in this study. The results showed highly conserved characteristics in structure, number of gene and intron, inverted repeat regions, and repeat sequences among the 29 cp genomes. Moreover, single-nucleotide polymorphism (SNP) loci with high polymorphism located at 17 fragments were selected as candidate SNP markers for future studies. In the phylogenetic tree, the cp genomes of tomatoes were clustered into two major clades, and the genetic relationship between *S. pimpinellifolium* and *S. lycopersicum* was very close. In addition, only *rps15* showed the highest average *K*_A_/*K*_S_ ratio in the analysis of adaptive evolution, which was strongly positively selected. It may be very important for the study of adaptive evolution and breeding of tomato. In general, this study provides valuable information for further study of phylogenetic relationships, evolution, germplasm identification, and molecular marker-assisted selection breeding of tomato.

## 1 Introduction

Cultivated tomato (*Solanum lycopersicum*) is an annual or perennial herb, which is a model system for Solanaceae and fruiting vegetables. The origin center of tomato is the Andes Mountains of South America, which is native to Peru, Ecuador, and other places in South America (Aoki et al., 2010). It has become one of the major cultivated vegetables in the world and contains rich nutrients. Significant differences in the nutrient composition of different varieties were detected by using high-performance liquid chromatography and electrochemical methods (Wang et al., 2023). Previous studies have shown that *S. pimpinellifolium* is the ancestor of cultivated tomatoes (Lin et al., 2014b), and *S. habrochaites* is an important wild relative of cultivated tomato, which has a variety of excellent disease resistance and stress resistance traits (Safari et al., 2021). Wild germplasm resources are widely used in modern tomato breeding. Therefore, it is necessary to identify the phylogenetic relationships among tomato germplasms by chloroplast (cp) genome sequencing, assembly, and annotation.

Chloroplast is the main place for energy conversion and photosynthesis of plants (Sadali et al., 2019). Compared with the large nuclear genome, the cp genome is smaller, and the copy number is more (Ashworth and Vanessa, 2017). The genome of cp is generally maternal inheritance; there is no problem of gene recombination (Li et al., 2014; Zhao et al., 2015). In recent years, the cp genome has been mainly used in phylogenetic, population genetics, and phylogeography studies (Wang et al., 2022b), in which plant phylogenetic analysis is the most basic cp genome analysis, which has been widely used in the phylogenetic study of the plant kingdom or one of the groups and to identify the relationships of angiosperm order, family, genus, interspecies, and intraspecies (Sun et al., 2020; Guo et al., 2021; Li et al., 2021; Xie et al., 2021; Liu et al., 2022; Raman et al., 2022; Wang et al., 2022a). The cp genome has unique advantages in plant phylogenetic studies. It had been favored in the study of plant molecular systematics because of its distinctive differences of molecular evolution rates in different regions, moderate nucleic acid replacement rates, and easily accessible sequences. The cp genome contains a large number of functional genes related to photosynthesis, gene expression, and other biosynthesis. Most of the cytoplasmically inherited traits are maternally inherited, and the development of cp molecular markers associated with maternally inherited traits has important applications in molecular marker-assisted selection breeding. Studies have shown that yellowing is the most common mutant phenotype in Chinese cabbage (*Brassica campestris* ssp. *pekinensis*), which is mostly inherited maternally. The mutated gene is the cp 16S small subunit protein gene *rps4*, and the presence of a single-nucleotide polymorphism (SNP) (A–C) with a mutation rate higher than 99% in the coding region of *rps4* resulted in the conversion of the RPS4 protein’s 193rd amino acid Val to Gly (Tang et al., 2018). Li et al. (2011) hypothesized that the upregulated expression of the RPS15a gene may be associated with the occurrence of the multi-ovary in wheat. Therefore, comparing cp genomes can identify some important variations in the evolution of species and provide a theoretical basis for the study of species relatedness and interspecific identification, while more cp molecular markers associated with maternal genetic traits can be developed for molecular marker-assisted selection breeding.

As an important family of plants, Solanaceae plants include many edible and medicinal plants (Martins da Silva et al., 2014), but there are only a few reports on the study of tomato cp genome. Daniell et al. (2006) conducted a comparative analysis of the complete cp genomes of wild potato (*Solanum bulbocastanum*), tomato (*S. lycopersicum*), tobacco (*Nicotiana tabacum*), and Atropa (*Atropa belladonna*). The results showed that deletions or insertions within some intergenic spacer regions result in less than 25% sequence identity. They can be used as an effective chloroplast marker in low-level phylogenetic studies. Chung et al. (2006) sequenced the cp genome of a cultivated potato (*Solanum tuberosum*) and compared it with the cp genome of six other Solanaceae plants, including *S. lycopersicum*. The results showed that there was a 241-bp deletion in the large single copy region (LSC) of cultivated potato, which could be used as a new method to identify cultivated potato and wild potato. Rachele et al. (2020) carried out a cp genome analysis with tomato as main material at first time. They sequenced seven cp genomes of cultivated accessions from Southern Italy and two wild species among the closest (*S. pimpinellifolium*) and most distantly related (*S. neorickii*) species to cultivated tomatoes. In total, 11 tomato cp genome sequences were retrieved in GenBank for comparative analysis with the abovementioned set. Finally, they found that *S. pimpinellifolium* was the nearest ancestor of all cultivated tomatoes. The local materials were closely related to other cultivated tomatoes. However, the SNP loci that could be used for future research were not screened, and adaptive evolution analysis was not carried out in the abovementioned study. Moreover, the research on the complete genome sequencing and analysis of tomato core germplasms from China and wild tomatoes’ cp genome has not been reported widely.

In view of the lack of classification and phylogenetic relationship between tomato interspecies and intraspecies, 29 tomato germplasms with different genetic backgrounds at home and abroad were screened from the core germplasms by our research group, and six wild resources were collected from Tomato Genetic Resource Center. The second-generation high-throughput sequencing technology was used to sequence the complete cp genome of tomato germplasms as well as assemble and annotate. Then, bioinformatics analysis was performed; a high-definition map of cp genome and a phylogenetic tree were constructed to clarify the phylogenetic relationship of tomato germplasms. Moreover, SNP loci with high polymorphism were selected as candidate SNP markers, and adaptive evolution analysis was conducted. This study will provide valuable information for further phylogenetic relationships, evolution, germplasm identification, and molecular marker-assisted selection breeding of tomato.

## 2 Materials and methods

### 2.1 Plant materials and genomic DNA isolation

In this study, 29 tomato core collections were selected and cultivated in the experimental farm of Ningxia University (**Table 1**). The fresh and healthy leaves of 29 tomato germplasms were collected, and then the leaf tissue samples were frozen fresh at -80°C until DNA extraction. The total genomic DNA was extracted using the New Plant Genome Extraction Kit (DP320) (Tiangen, Beijing, China) according to the manufacturer’s instructions. The purity and integrity of the genomic DNA samples were identified using 1% agarose gel electrophoresis. The concentration of genomic DNA samples was measured using a NanoDrop 2000C spectrophotometer (Thermo Scientific; Waltham, MA, USA).

**Table 1:** Material sources of 29 tomato germplasms.

Sample name	Material name	Taxon	GenBank accession number	Location	Germplasm type	Mature fruit color	Fruit types
A1	LA3432	*S. lycopersicum*	OQ473528	USA	Cultivar	Red	Big fruit
A2	LA3474	*S. lycopersicum*	OQ473535	USA	Cultivar	Red	Big fruit
A3	LA2822	*S. lycopersicum*	OQ473542	USA	Cultivar	Red	Big fruit
A4	LA1969	*S. chilense*	OQ473544	Peru	Wild species	Red	Small fruit
A5	LA1269	*S. pimpinellifolium*	OQ473545	Peru	Wild species	Red	Small fruit
A6	20CL1036	*S. lycopersicum*	OQ473546	China	Cultivar	Red	Big fruit
A7	21CL0625	*S. lycopersicum*	OQ473547	China	Cultivar	Yellow	Cherry fruit
A8	21CL0668	*S. lycopersicum*	OQ473548	China	Cultivar	Pink	Big fruit
A9	21CL1999	*S. lycopersicum*	OQ473549	China	Cultivar	Red	Big fruit
A10	20CL2110	*S. lycopersicum*	OQ473521	China	Cultivar	Pink	Big fruit
A11	21CL0579	*S. lycopersicum*	OQ473522	China	Cultivar	Yellow	Cherry fruit
A12	21CL0031	*S. lycopersicum*	OQ473523	China	Cultivar	Pink	Cherry fruit
A14	21CL0381	*S. lycopersicum*	OQ473524	China	Cultivar	Red	Cherry fruit
A15	21CL0033	*S. lycopersicum*	OQ473525	China	Cultivar	Pink	Cherry fruit
A16	21CL1205	*S. lycopersicum*	OQ473526	China	Cultivar	Pink	Big fruit
A17	LA1245	*S. pimpinellifolium*	OQ473527	Ecuador	Wild species	Pink	Small fruit
A21	LA2399	*S. lycopersicum*	OQ473529	USA	Cultivar	Red	Big fruit
A23	D61825R	*S. lycopersicum*	OQ473530	China	Cultivar	Red	Big fruit
A24	D62333R	*S. lycopersicum*	OQ473531	China	Cultivar	Red	Big fruit
A27	D62140P	*S. lycopersicum*	OQ473532	China	Cultivar	Pink	Big fruit
A28	D62180P	*S. lycopersicum*	OQ473533	China	Cultivar	Pink	Big fruit
A29	D61867G	*S. lycopersicum*	OQ473534	China	Cultivar	Green	Big fruit
A33	Micro-tom	*S. lycopersicum*	OQ473536	USA	Cultivar	Red	Small fruit
A34	Moneymaker	*S. lycopersicum*	OQ473537	USA	Cultivar	Pink	Big fruit
A35	21CL2625	*S. lycopersicum*	OQ473538	China	Cultivar	Orange	Big fruit
A36	21CL2646	*S. lycopersicum*	OQ473539	China	Cultivar	Red	Big fruit
A38	LA2329	*S. habrochaites*	OQ473540	Peru	Wild species	Green	Small fruit
A39	LA2809	*S. peruvianum*	OQ473541	Peru	Wild species	Red	Small fruit
A41	62442	*S. habrochaites*	OQ473543	Peru	Wild species	Green	Small fruit

### 2.2 Tomato genome sequencing, assembly, and annotation

The high-throughput sequencing of 29 tomato germplasms was completed by Berry Genomics Co., Ltd. After the DNA samples were qualified, the genomic DNA was randomly cut into 350-bp fragments by enzyme digestion. After terminal repair and poly A addition, the sequencing adapters were connected at both ends of the fragments. Lastly, the libraries were analyzed for size distribution using agarose gels and were quantified using real-time PCR. The clustering of the index-coded samples was performed on a cBot Cluster Generation System using Novaseq 6000 S4 Reagent Kit (Illumina) according to the manufacturer’s instructions. After cluster generation, the DNA libraries were sequenced on Illumina NovaSeq 6000 platform, and 150-bp paired-end reads were generated.

### 2.3 Analyses of repetitive sequences

Repetitive sequences in the cp genome play a critical role in genome evolution and rearrangements. The simple sequence repeats (SSR) motifs were analyzed in the cp genome of 29 tomato germplasms using MISA v2.1 (Raman et al., 2022). The minimum repeat thresholds of 10, six, five, five, five, and five are for mono-, di-, tri-, tetra-, penta-, and hexa-nucleotide SSRs, respectively. Tandem repeats were analyzed using the TRF (Benson, 1999) software with default parameters (Liu et al., 2022). In addition, oligonucleotide repeat analysis of four types of repeats in the cp genome was carried out. The forward, reverse, complement, and palindromic repeats were detected using REPuter online software (Kurtz et al., 2001) with a minimum repeat size of 30 bp and 90% sequence identity (Hamming distance of 3).

### 2.4 Boundary regions and chloroplast genome sequence comparison

The connecting regions of IR-LSC and IR-SSC in the cp genomes of 29 tomato germplasms were compared by using IRscope online software (https://irscope.shinyapps.io/irapp/) (Amiryousefi et al., 2018). The mVISTA online software (http://genome.lbl.gov/vista/mvista/submit.shtml) was used to compare the cp genomics of 29 tomato germplasms (Frazer et al., 2004). The comparative analysis was carried out by using the shuffle-LAGAN mode in mVISTA, and the sequence alignment was visualized in an mVISTA plot. The size of the sliding window is set to 100, and the default values for minimum and maximum Y are 50% to 100%. Mauve V2.4.0 was used to compare the cp genomes of 29 tomato germplasms to determine the collinearity of cp genome structure and identify possible rearrangements (Katoh et al., 2019). We also calculated the nucleotide diversity (Pi) of 80 protein-coding genes and intergenic spacer regions among the 29 tomato germplasms (Raman et al., 2022).

### 2.5 Analysis of phylogenetic relationship

The coding sequences, intergenomic sequences, and the complete cp genomes of 29 tomato germplasms were selected to construct a phylogenetic tree, and *Solanum bulbocastanum* DQ347958 was selected as the outgroup. To analyze the phylogenetic relationship of tomato, alignments were used to construct the phylogenetic trees using the maximum likelihood (ML) method implemented in RAxML v8.2.12 with 1,000 bootstrap replicates (Nguyen et al., 2015).

### 2.6 Analysis of substitution rate

In this study, the complete cp genomes of 29 tomato germplasms were compared. By extracting the same specific protein-encoded DNA sequence and translating it into a protein sequence, protein sequence alignment was performed using ParaAT3.0 software, and then the nucleic acid alignment result corresponding to a codon was translated back according to the protein alignment result. After the homologous sequence alignment, KaKs_Calculator 3.0 software was used to calculate the synonymous (*K*_S_) and nonsynonymous (*K*_A_) substitution rates and *K*_A_/*K*_S_ ratios (Zhang, 2022).

## 3 Results

### 3.1 General features of the tomato chloroplast genome

**Figure 1:**
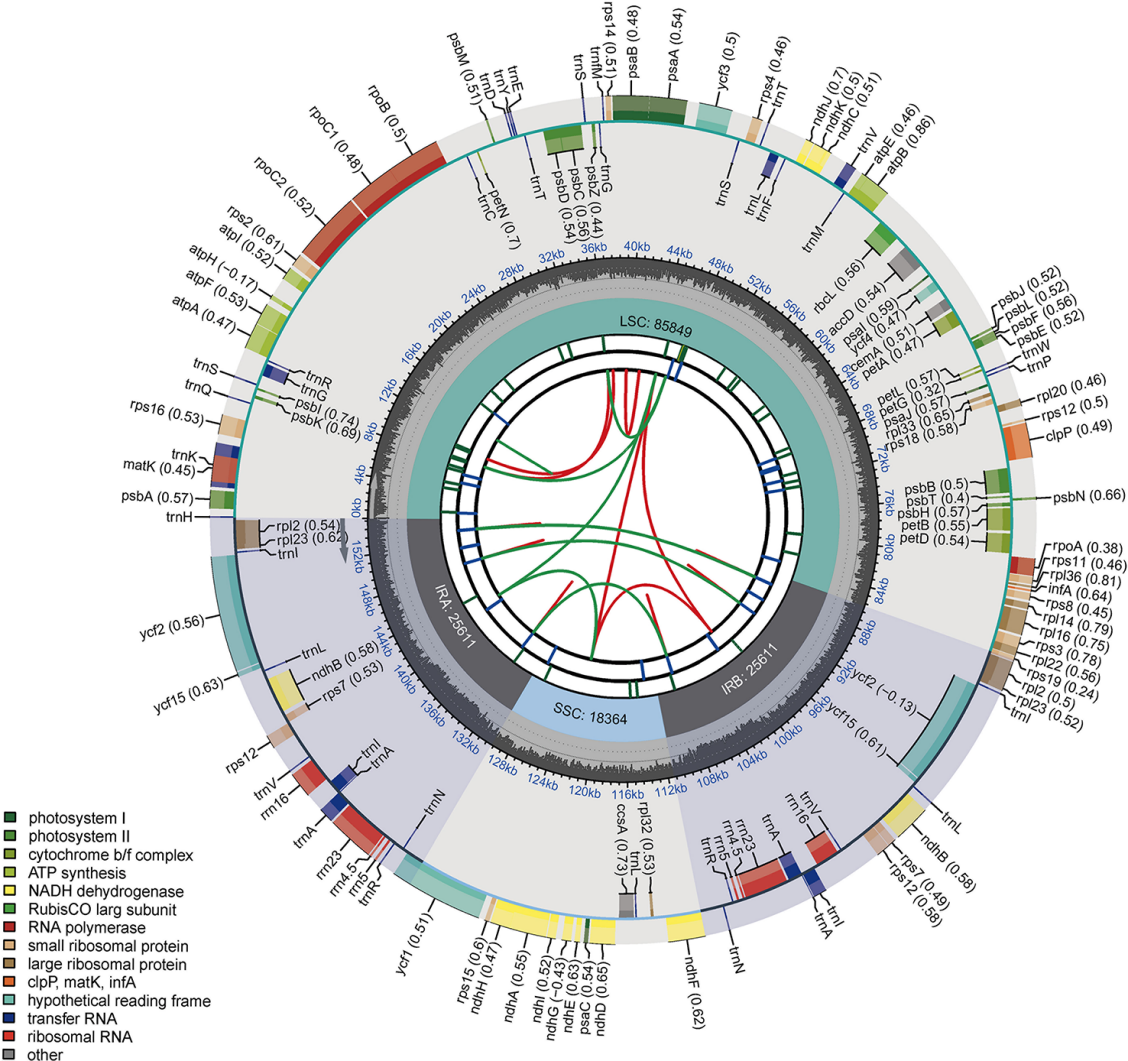
Circular chloroplast genome map of tomato. Genes drawn outside the circle are transcribed clockwise, and those inside are counterclockwise. Genes belonging to different functional groups are color-coded. The darker gray in the inner circle shows the GC content, while the lighter gray shows the AT content.

The raw data were deposited in the Sequence Read Archive under BioProject accession number PRJNA936910. In this study, the cp genomes of 29 tomato germplasms were sequenced and characterized. The final cp genomes were assembled, annotated, and submitted to GenBank. The complete cp genomes of the 29 tomato germplasms ranged from 155,257 to 155,461 bp in length. Each cp genome was made up of three distinct regions (**Figure 1**). The length of the IR region ranged from 25,594 to 25,612 bp. The length of the LSC region ranged from 85,688 bp (A39 and A41) to 85,875 bp (A4), and the length of the SSC region ranged from 18,355 bp (A38, A39, and A41) to 18,375 bp (A4). Among 29 tomato germplasms, the total GC content of the cp genomes of A38 was the lowest (37.84%), while that of A5 was the highest (37.87%). The total GC content of the cp genome of each of the remaining 27 germplasms was 37.86% (**Supplementary Table S1**).

In addition, the number of genes and introns were highly conserved, and the same suite of protein-coding genes, ribosomal RNA (rRNA) genes, and transfer RNA (tRNA) genes was found in all taxa. Each cp genome included 113 unique genes, which contained 80 protein-coding genes, 29 tRNA genes, and four rRNA genes (**Supplementary Table S2**). In 113 genes, 18 genes with introns were identified. Among them, nine protein-coding (*atpF*, *ndhA*, *ndhB*, *petB*, *petD*, *rpl16*, *rpl2*, *rpoC1*, and *rps16*) and six tRNA genes (*trnA-UGC*, *trnG-GCC*, *trnI-GAU*, *trnK-UUU*, *trnL-UAA* and *trnV-UAC*) contained one intron, whereas *clpP*, *rps12*, and *ycf3* contained two introns.

### 3.2 Inverted repeat region contraction and expansion in chloroplast genomes

**Figure 2:**
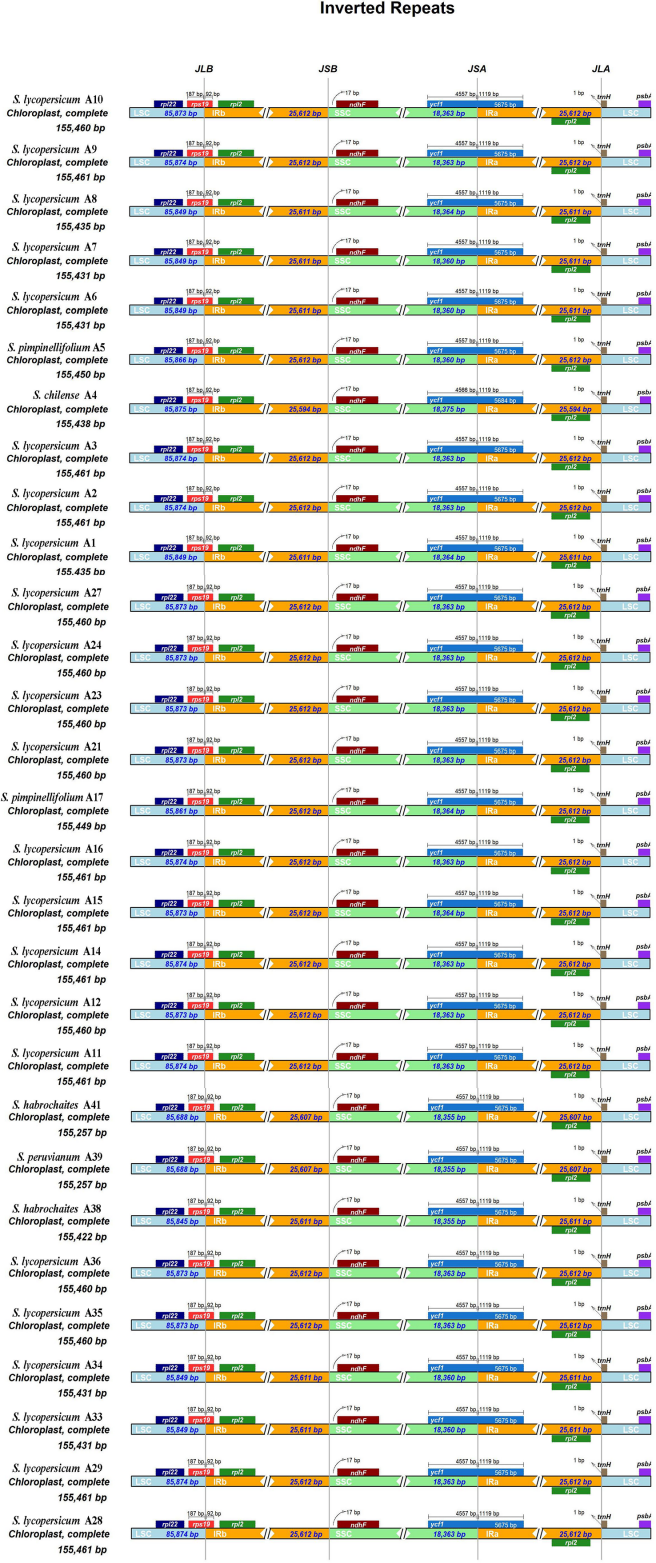
Comparison of the borders of large single copy (LSC), small single copy (SSC), and inverted repeat (IR) regions among 29 tomato germplasm chloroplast genomes. JLB line indicates the border between LSC and IRb, JSB line indicates the border between SSC and IRb, JSA line indicates the border between SSC and IRa, and JLA line indicates the border between LSC and IRa.

There were four borders between LSC, IRb, SSC, and IRa in the cp genome: LSC/IRb border, IRb/SSC border, SSC/IRa border, and IRa/LSC border. The borders of the 29 tomato germplasm cp genomes were compared (**Figure 2**). The four borders were conservative. The *rpl22* gene was present in the LSC region, and *rpl2* gene existed entirely in the IR region. Additionally, the *rps19* gene straddled the boundary of the LSC/IRb regions. The *ndhF* gene was located at the IRb/SSC border, and the distance between *ndhF* and the JSB line was 17 bp. The *ycf1* gene was observed at the JSA line, which straddled the boundary of the SSC/IRa regions. The *trnH* noncoding gene was located on the right side of the JLA line with a distance of 1 bp. In addition, the *psbA* gene existed in the LSC.

### 3.3 Codon usage in tomato chloroplast genomes

The amino acid frequency, codon usage, and relative synonymous codon usage (RSCU) of 80 protein-coding regions in 29 tomato germplasms were analyzed using Codon W. The RSCU values ranged from 63.99 to 64.03, the number of codons ranged from 23,010 to 23,016 in 29 tomato germplasms, and the number of amino acids ranged from 22,930 to 22,936. Of these amino acids, leucine (2,439–2,445 codons) was the most abundant amino acid, with a frequency of 10.63%–10.66%, while the frequency of cysteine (257 codons) was 1.12%. However, the most often used codon was ATT (encoding isoleucine), and the least used was TGA (termination codon). Almost all the amino acids had more than one synonymous codon; the exceptions were methionine and tryptophan. Furthermore, 62 codons displayed RSCU values exceeding 1.00. ATG and TGG, encoding methionine and tryptophan separately, exhibited no bias (RSCU = 1.00) (**Supplementary Table S3**). Moreover, three types of starting codons (ATG, GTG, and ACG) were detected in 80 protein-coding genes. Most genes used ATG as the starting codon. TAA, TAG, and TGA were present as stop codons in these genes. The most often used stop codon was TAA at 52.5%, followed by TAG (26.25%) and TGA (21.25%).

### 3.4 Comparative analysis of the repeat sequences in tomato genomes

**Figure 3:**
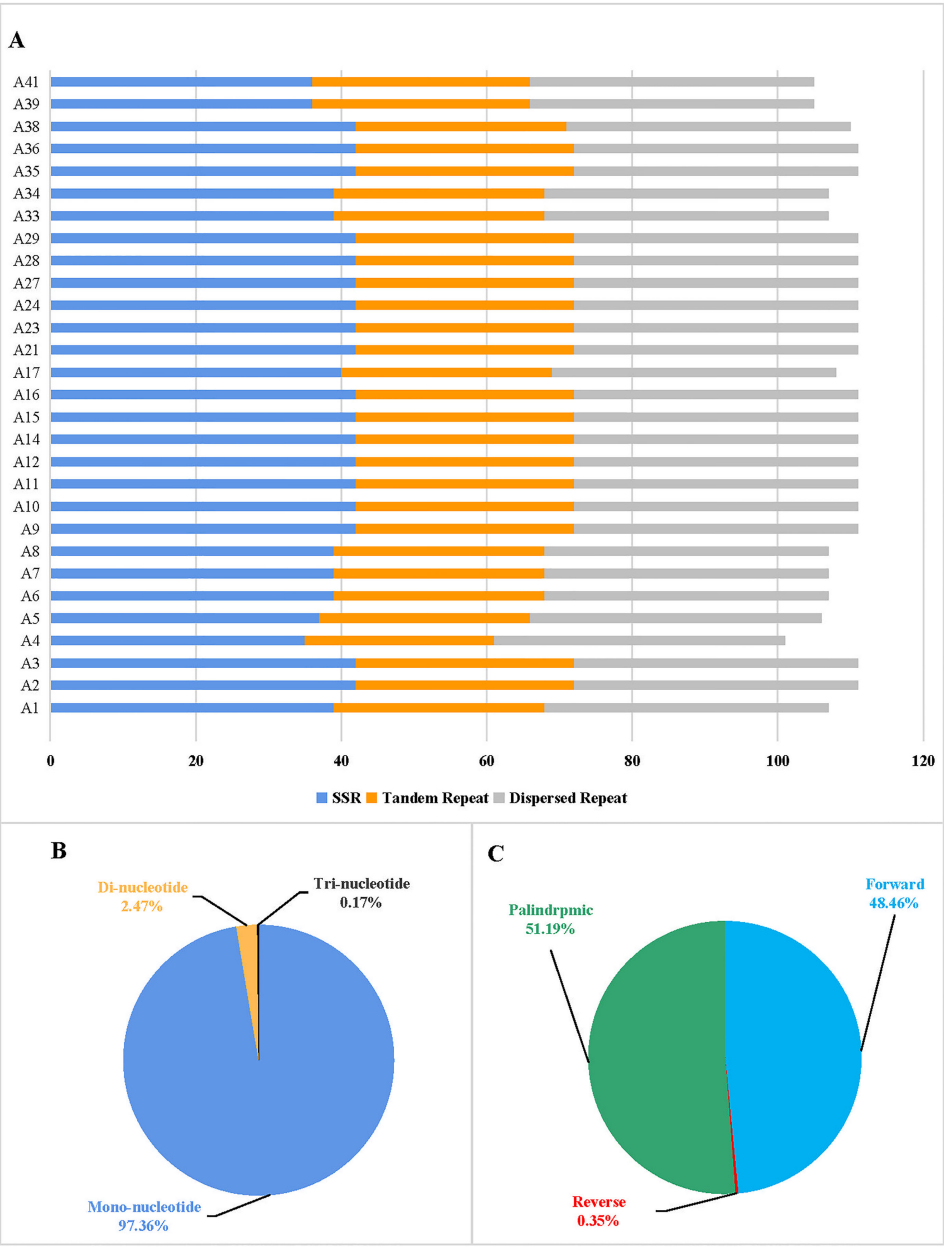
Histogram showing the number of repeats in 29 tomato germplasm chloroplast genomes. **(A)** Distribution of simple sequence repeats (SSRs), tandem repeats, and dispersed repeats in 29 tomato germplasms. **(B)** Proportion of different SSR repeat types. **(C)** Proportion of different dispersed repeat types.

In this study, the repeat sequences of the cp genomes of 29 tomato germplasms were analyzed. The distribution of SSRs, tandem repeats, and dispersed repeats differs among the 29 tomato germplasms (**Figure 3A**). Furthermore, we identified the total number of SSRs per cp genome that ranges from 36 to 42. Mononucleotides were the most frequent in the SSRs, with a distribution of 97.36%, followed by dinucleotide and trinucleotides at 2.47% and 0.17%, respectively, in the 29 tomato germplasms (**Figure 3B**). There was a dinucleotide with a predominant motif of TA per cp genome in 29 tomato germplasms. Trinucleotides were absent in the cp genome of most tomato germplasms, except for a TTA motif in A4 and a TAA motif in A38. Moreover, the distribution of tandem repeats in the tomato cp genomes ranges from 26 to 30.

In addition to SSRs and tandem repeats, a dispersed repeat analysis of four types of repeats in the cp genome, including forward (F), reverse (R), palindromic (P), and complementary (C), was performed using REPuter. The total number of 39 dispersed repeats was identified in each cp genome of most tomato germplasms, while A4 and A5 had 40 dispersed repeats. Among the tomato cp genomes, forward repeats and palindromic repeats were the most common, accounting for 48.46% and 51.19%, respectively (**Figure 3C**). Only A4, A5, A39, and A41 had one reverse repeat, respectively. Complement repeats were not observed in the cp genomes of 29 tomato germplasms (**Supplementary Table S4**).

### 3.5 Comparative chloroplast genome analysis

Multiple alignments of 29 tomato cp genomes were conducted using the online software platform mVISTA. A comparison of overall sequence variation showed that the tomato cp genome is highly conserved. Only *ycf1* open reading frame had a divergence in the coding region (**Supplementary Figure S1**). Subsequently, Mauve was used to identify the local collinear blocks (LCBs) of 29 tomato germplasm cp genomes (**Supplementary Figure S2**). These germplasms showed a consistent sequential order in all genes. The LCBs of all cp genomes showed a relatively high conservation with no gene rearrangement.

**Figure 4:**
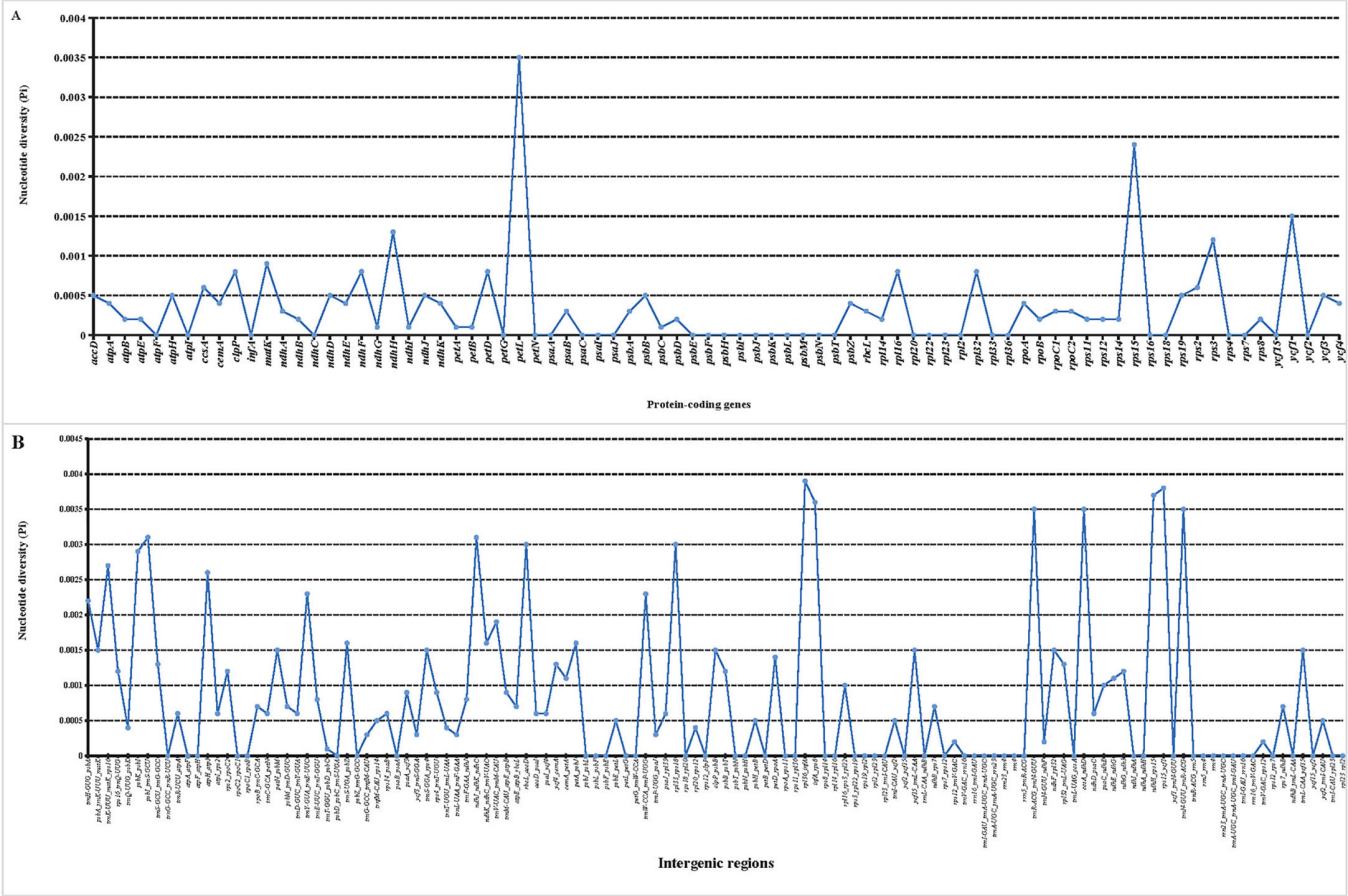
Nucleotide diversity (Pi) value in 29 tomato germplasm chloroplast genomes. **(A)** Pi value of protein-coding genes. **(B)** Pi value of intergenic spacer regions.

In addition, the nucleotide diversity (Pi) of 80 protein genes and intergenic spacer regions in the tomato cp genomes was calculated. A total of 80 genes were related to transcription, translation, and photosynthetic processes, with low variation (**Figure 4A**). Among these 80 genes, *petL* had the highest Pi value (0.0035), followed by *rps15* (0.0024) and *ycf1* (0.0015), showing obvious divergences. Meanwhile, 32 genes had a Pi value of 0. However, obvious divergences were detected in the intergenic spacer regions of *psbI-trnS-GCU*, *rpl36-infA*, *infA-rps8*, *trnR-ACG-trnN-GUU*, *ccsA-ndhD*, *ndhH-rps15*, *rps15-ycf1*, and *trnN-GUU-trnR-ACG* (**Figure 4B**). The Pi value of these six genes was above 0.003. In addition, SNP/MNP loci with high polymorphism located at 17 fragments were selected as candidate SNP markers for future studies (**Table 2**). Among the screened SNP markers, those localized to fragments of the *ndhH* gene and the *ndhK-ndhC-trnV-UAC* intergenic spacer regions could be used for interspecific identification.

**Table 2:** Candidate polymorphic DNA markers among chloroplast genomes of 29 tomato germplasms.

No.	Position*	Polymorphic Type	Variant	Location
1	59,407-59,775	SNP	A/T, G/A, A/G, A/C, G/A	*accD*
2	111,655-113,611	SNP	G/T, A/G, C/T, G/A, G/A, C/A, C/G, G/A, C/T, C/T, A/G, C/T, A/C, C/A, C/A, G/A	*ndhF*
3	123,639-124,359	SNP	A/G, C/G, A/C, C/T, C/T, T/C, G/A	*ndhH*
4	84,531-85,109	SNP	A/G, G/A, A/G, C/T, G/A, C/A	*rps3*
5	17,372-18,226	SNP	C/T, G/A, T/C, G/A, A/C	*rpoC2*
6	24,194-24,416	SNP	G/T, C/T, C/A	*rpoB*
7	124,633-124,834	SNP	C/T, G/A, G/A, G/A	*rps15*
8	120,153-120,479	SNP	C/A, A/C, T/G	*ndhG-ndhI*
9	51,876-52,585	SNP	T/A, T/A, G/T, G/T, A/G, C/T, G/T, G/T	*ndhK-ndhC-trnV-UAC*
10	64,959-65,070	SNP	TA/AC, C/T, A/T, A/G, G/A, TG/AA, A/G, C/T, TT/CA, T/C, C/T, A/T, A/G, T/A, G/A, G/A	*petA-psbJ*
11	16,544-16,547	MNP	TTTC/AAAA	*rps2-rpoC2*
12	6330-7031	SNP/MNP	T/G, AT/GT, T/G, C/A, TTG/AAA	*rps16-trnQ-UUG*
13	58,197-58,228	SNP/MNP	A/T, T/C, C/T, TAGT/ACTA, A/G, G/A	*rbcL-accD*
14	49,422-49,995	SNP	C/A, C/A, A/G, G/A	*trnF-GAA-ndhJ*
15	208-437	SNP	G/A, A/G, T/G, C/A, G/A, G/A	*trnH-GUG-psbA*
16	109,378-109,612	MNP	CTTT/AAAG, AAA/TTT	*trnN-GUU-trnR-ACG*
17	131,673-131,907	MNP	TTT/AAA, AAAG/CTTT	*trnR-ACG-trnN-GUU*

*Position is based on the alignment file. SNP, single nucleotide polymorphism; MNP, multiple nucleotide polymorphism.

### 3.6 Phylogenetic relationship analysis

**Figure 5:**
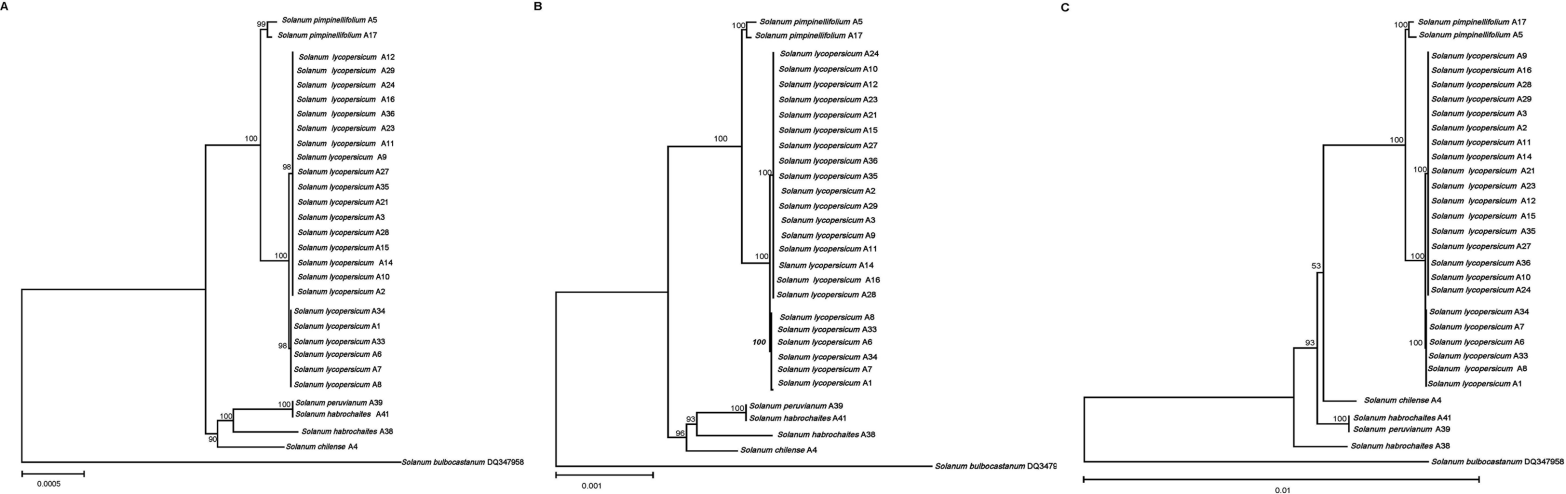
Maximum likelihood phylogenetic tree of 29 tomato germplasms. **(A)** Based on protein-coding sequences. **(B)** Based on complete chloroplast genomes. **(C)** Based on intergenomic sequences. Values above the branches represent the maximum likelihood bootstrap value. .

In order to study the phylogenetic relationship among different tomato germplasms, ML phylogenetic trees were constructed using coding sequences, intergenomic sequences, and the complete cp genomes of 29 tomato germplasms (**Figure 5**). *Solanum bulbocastanum* DQ347958 selected as outgroups were retrieved from NCBI. In the phylogenetic tree, the tomatoes were clustered into two major clades based on coding sequences and the complete cp genomes, with one clade comprising all cultivated tomato and *S. pimpinellifolium* and the other clade containing the remaining four wild tomatoes. Furthermore, the first clade could be further divided into two minor clades, with all cultivated tomato clustered into the same minor clade and the other contained two *S. pimpinellifolium*, indicating a relatively closer interrelationship between cultivated tomato and *S. pimpinellifolium*. The second clade could also be divided into two minor clades, with one minor clade comprising only *S. chilense* and the other including *S. habrochaites* and *S. peruvianum* (**Figures 5A, B**). However, the phylogenetic results based on intergenomic sequences are different from the abovementioned results based on coding sequences and the complete cp genomes. Based on intergenomic sequences, the tomatoes were clustered into two major clades. The first can be further divided into two minor clades, with all cultivated tomatoes and two wild tomatoes (two *S. pimpinellifolium* and one *S. chilense*, respectively) clustered into the first minor clade and the other minor clade contained *S. habrochaites* A41 and *S. peruvianum* A39. *S. habrochaites* A38 was clustered separately as the second major clade. It indicated that *S. chilense* was more closely related to cultivated tomatoes than *S. habrochaites* and *S. peruvianum*.

### 3.7 Adaptive evolution analysis

**Figure 6:**
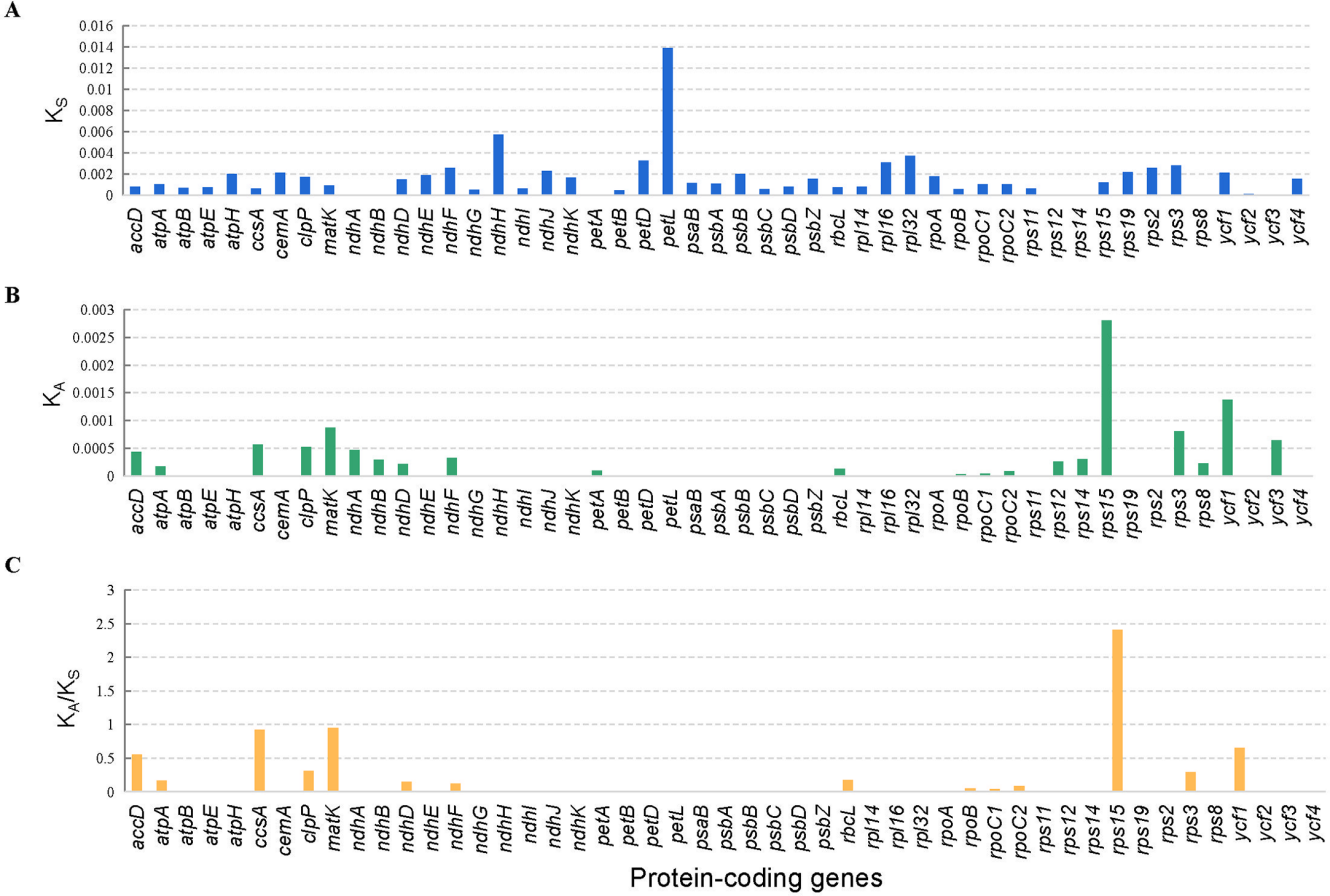
Selective pressure of 49 protein-coding genes in 29 tomato cp genomes. **(A)** *K*_S_, rate of synonymous substitution. **(B)** *K*_A_, rate of nonsynonymous substitution. **(C)** *K*_A_/*K*_S_, rate of non-synonymous *vs*. synonymous substitution.

In total, 49 protein-coding genes of all the 29 tomato cp genomes were used for the analysis of synonymous (*K*_S_) and non-synonymous (*K*_A_) substitution rates. The results showed that most protein-coding genes have relatively low average *K*_S_ values (<0.008), except the *petL* genes (**Figure 6B**). In the same way, the average *K*_A_ values of most protein-coding genes were comparatively low (<0.0015), except the *rps15* and *ycf1* genes (**Figure 6A**). The average *K*_A_/*K*_S_ ratio of *rps15* gene was the highest (2.41). Furthermore, the *K*_A_/*K*_S_ ratios of all the protein-coding genes ranged from 0 to 2.41, with an average ratio of only 0.14 (**Figure 6C**).

## 4 Discussion

Due to the slow evolution of plant cp genome, it has been used for plant classification and molecular evolution research. The study of species identification and phylogenetic evolution based on cp genome is a development trend of plant taxonomy biology, which has attracted more and more attention and recognition from researchers (Nock et al., 2015). The genetic background of tomato is more and more narrow due to the loss of genetic diversity, selfing characteristics, and long-term domestication in the process of tomato spreading from the origin to the world (Doebley et al., 2006). However, tomato wild relatives have a rich genetic diversity and are an inexhaustible gene library for the genetic improvement of tomato. So far, there are few reports on the comparative study of tomato cp genome. Rachele et al. (2020) compared and analyzed nine tomato cp genomes obtained by sequencing and 11 tomato cp genomes retrieved from GenBank. In this study, the cp genome sequences of 29 tomato germplasms were sequenced and compared, which also showed highly conserved characteristics in structure, number of gene and intron, inverted repeat regions, which was similar to the study of Rachele et al. (2020).

Repeats in the cp genome play an important role in genome evolution and rearrangements (Wang et al., 2022a). SSR can be used as a molecular marker and used in population genetics research because of its high polymorphism (Zhang et al., 2012; Zhao et al., 2015). However, among 29 tomato cp genomes, the number of mononucleotide repeats was the largest, accounting for the majority of all SSRs (97.36%). Therefore, polymorphisms existed in the SSRs of the tomato cp genome, but their repeat numbers were relatively conservative. Whether they could be used as molecular markers independently for population genetic analysis needs to be further validated. However, trinucleotide repeats were only present in A4 (*S. chilense*) and A38 (*S. habrochaites*) (TTA and TAA motifs, respectively), which may be used as SSR candidate markers for the study of subsequent germplasm identification. Moreover, the tandem repeats and dispersed repeats of tomato cp genome were relatively conservative. Previous studies have shown that repeats have a great influence on insertion and substitution, which can increase the genetic diversity of biological populations (Klein and O’Neill, 2018). The existence and abundance of cp repetitive sequences may also be related to multiple phylogenetic signals (Zhang et al., 2011; Wang et al., 2016), while the repetitive sequences on the tomato cp genome are relatively conservative, and whether it is related to phylogenetic signals needs further study.

In addition, 17 SNP/MNP loci with high polymorphism were selected as candidate SNP markers in this study. SNP molecular markers play an important role in tomato breeding, mainly in the identification of genetic relationship and genetic diversity of germplasm resources, the construction of genetic linkage map, the localization of target genes, and the identification of variety purity and molecular marker-assisted selection breeding (Kim et al., 2021). At present, the development of chloroplast SNP markers has been applied to many plants—for example, Cesare et al. (2010) identified six cp markers containing both cp SSRs and SNPs, and these SNP markers can distinguish most *Miscanthus* species and detect intraspecific variations, which can be used for breeding purposes. Lin et al. (2014a) developed four cp SNP molecular markers to distinguish soybean male sterile lines and maintainer lines and also to distinguish hybrids and soybean maintainer lines. The highly polymorphic SNP loci screened in this study were located at the regions of *ndhG-ndhI*, *ndhK-ndhC*, *petA-psbJ*, *rps2-rpoC2*, *rps16-trnQ-UUG*, *rbcL-accD*, *trnF-GAA-ndhJ*, *trnH-GUG-psbA*, *trnN-GUU-trnR-ACG*, and *trnR-ACG-trnN-GUU* and in *accD*, *ndhF*, *ndhH*, *rps3*, *rpoC2*, *rpoB*, and *rps15* genes. They will be developed as candidate cp genome SNP molecular markers for future studies. Among the screened SNP markers, those localized to segments of the *ndhH* gene and the *ndhK-ndhC-trnV-UAC* gene spacer region could be used for interspecific identification. The other developed SNP marker can be used to analyze genetic diversity and population structure at the cp genome level and to develop functional markers associated with traits such as male sterility, which has an important application value in tomato germplasm identification and molecular marker-assisted selection breeding.

The cultivated tomatoes were domesticated from wild tomatoes. The fruit weight of modern cultivated tomato is more than 100 times that of its ancestors. In order to reveal the secret of tomatoes from small to large, Lin et al. (2014b) used genome-wide variation group data to analyze the phylogeny and population structure of tomatoes, and they found that the tomato population was divided into three subgroups, namely, *S. pimpinellifolium*, *S. lycopersicum* var. *cerasiforme*, and large-fruit cultivated tomato (*S. lycopersicum*). Combined with abundant phenotypic data and population genetics analysis, it was proved that wild tomato (*S. pimpinellifolium*) evolved into cherry tomato (*S. lycopersicum* var. *cerasiforme*) and finally formed a two-step artificial selection process of large-fruit cultivated tomato—namely, domestication and improvement. The comparative analysis of the 29 tomato germplasm cp genomes sequenced in this work allowed the phylogenetic relationships among wild and cultivated germplasms to be defined and also indicated that the genetic relationship between *S. pimpinellifolium* and *S. lycopersicum* was very close—that is, *S. pimpinellifolium* may be the ancestor of cultivated tomatoes—which was consistent with the previous research results. Notably, the phylogenetic relationships based on coding sequences and the complete cp genomes were consistent with the results based on traditional botanical classification (Zhao, 2012). However, the phylogenetic relationships based on intergenomic sequences were different from the results based on coding sequences and the complete cp genomes and traditional botanical classification, which may be due to the fact that intergenomic sequences contain more variability. The phylogenetic relationships based on intergenomic sequences show that *S. chilense* is more closely related to cultivated tomatoes and *S. pimpinellifolium* than *S. habrochaites* and *S. peruvianum*.

The *K*_A_/*K*_S_ ratio is associated with gene adaptive evolution, such as positive selection and purity selection (Raman et al., 2022). In general, non-synonymous substitutions can cause amino acid changes, resulting in changes in protein conformation and function. Therefore, it will cause adaptive evolution and bring the advantages or disadvantages of natural selection. Synonymous substitution does not change the composition of the protein, so it is not affected by natural selection; then, Ks can reflect the background base substitution rate of the evolutionary process (Hurst, 2002). The *K*_A_/*K*_S_ ratio can explain the type of selection of this gene. When *K*_A_/*K*_S_ <<1, the gene is selected by purification. The *K*_A_/*K*_S_ of most genes is far less than 1 because generally non-synonymous substitutions bring evolutionary disadvantages, and only a few cases will result in evolutionary advantages. When *K*_A_/*K*_S_ >>1, the genes are strongly positively selected, and these genes are rapidly evolving recently and are of great significance for the evolution of species. We can screen some genes for further functional studies according to the *K*_A_/*K*_S_ ratio, which has been widely applied to the field of molecular evolution (Navarro and Barton, 2003). In this study, the analysis results of synonymous (*K*_S_) and non-synonymous (*K*_A_) substitution rates showed that *petL* genes have relatively high average *K*_S_ values. The *rps15* and *ycf1* genes have relatively high average *K*_A_. Only *rps15* showed the highest average *K*_A_/*K*_S_ ratio of 2.41, which was strongly positively selected. It may be a gene that is rapidly evolving recently, and its function can be further studied. It may be very important for the study of adaptive evolution and breeding of tomato.

## 5 Conclusion

In this study, the latest sequencing results of the chloroplast genomes of 29 tomato germplasms were reported and compared. Genome annotation and comparative analysis showed that each chloroplast genome was a typical tetragonal structure. The 29 chloroplast genomes are highly conserved in terms of structure, gene and intron number, IR region, and repeat sequences. In addition, we had screened SNP/MNP loci with high polymorphism located at 17 fragments of the regions of *ndhG-ndhI*, *ndhK-ndhC*, *petA-psbJ*, *rps2-rpoC2*, *rps16-trnQ-UUG*, *rbcL-accD*, *trnF-GAA-ndhJ*, *trnH-GUG-psbA*, *trnN-GUU-trnR-ACG*, and *trnR-ACG-trnN-GUU* and in *accD*, *ndhF*, *ndhH*, *rps3*, *rpoC2*, *rpoB*, and *rps15* genes in the cp genomes of 29 tomato germplasms, which will be used as candidate SNP markers in future studies. In the phylogenetic tree, the cp genomes of tomatoes were clustered into two major clades, and the genetic relationship between *S. pimpinellifolium* and *S. lycopersicum* was very close. Moreover, in the analysis of adaptive evolution, only *rps15* showed the highest average *K*_A_/*K*_S_ ratio of 2.4, which was strongly positively selected. In general, this study will provide valuable information for further study of phylogenetic relationships, germplasm identification, and molecular marker-assisted selection breeding of tomato.

## Data availability statement

The datasets presented in this study can be found in online repositories. The names of the repository/repositories and accession number(s) can be found in the article/**Supplementary Material**.

## Author contributions

XW and JL conceived and designed the study. FZ, LS, and LW collected and identified the plant materials. SB and ZZ performed the experiments and analyzed the data. XW and SB wrote the manuscript. XW, WY, MG, GC, and YG revised the manuscript. All authors contributed to the article and approved the submitted version.
