# Supplementary figures and images for: Comparative analysis of chloroplast genomes of 29 tomato germplasms: genome structures, phylogenetic relationships, and adaptive evolution
Source: Front Plant Sci. 2023 May 9;14:1179009. doi: 10.3389/fpls.2023.1179009 (PMC10203424; doi:10.3389/fpls.2023.1179009)

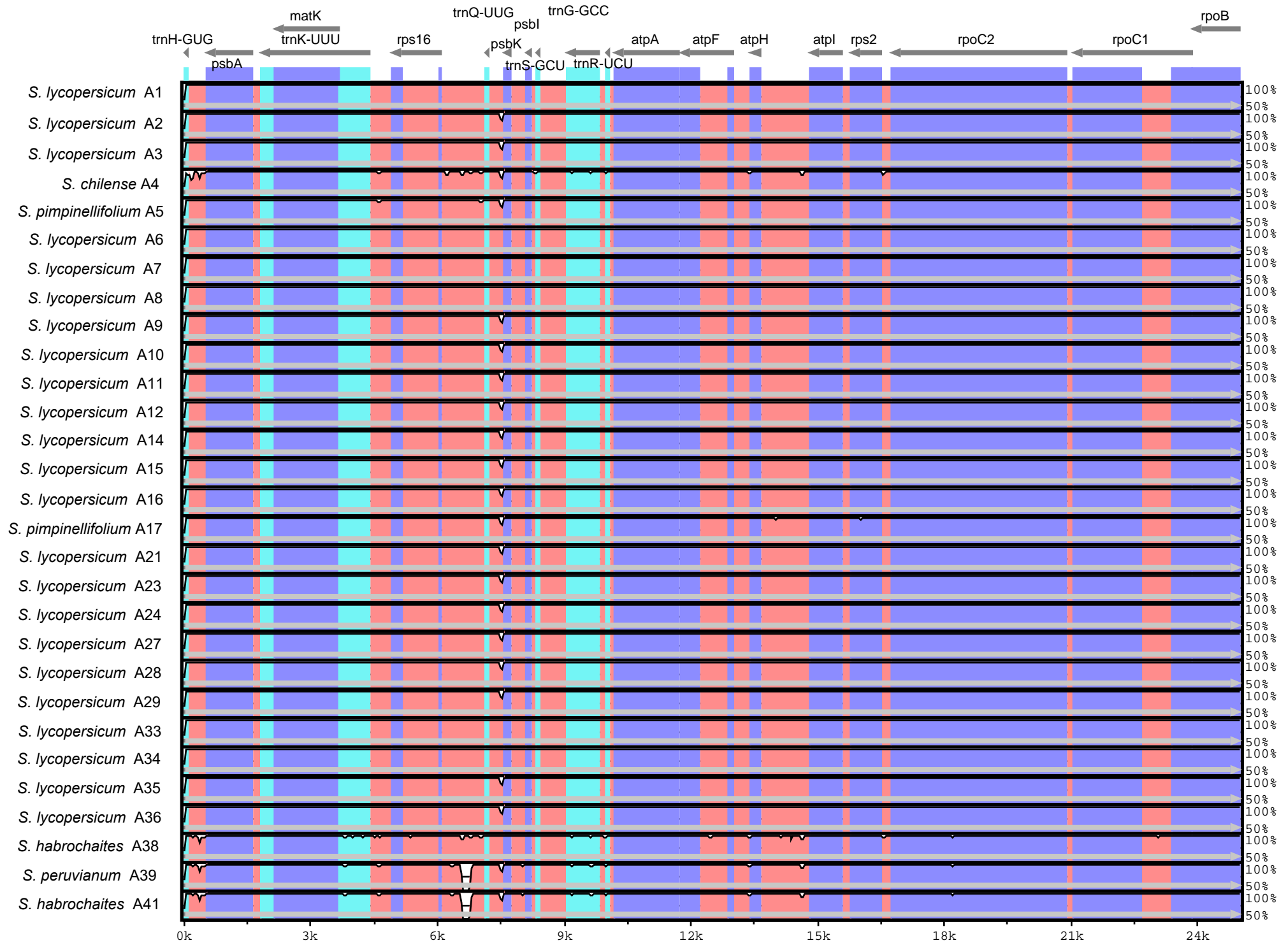

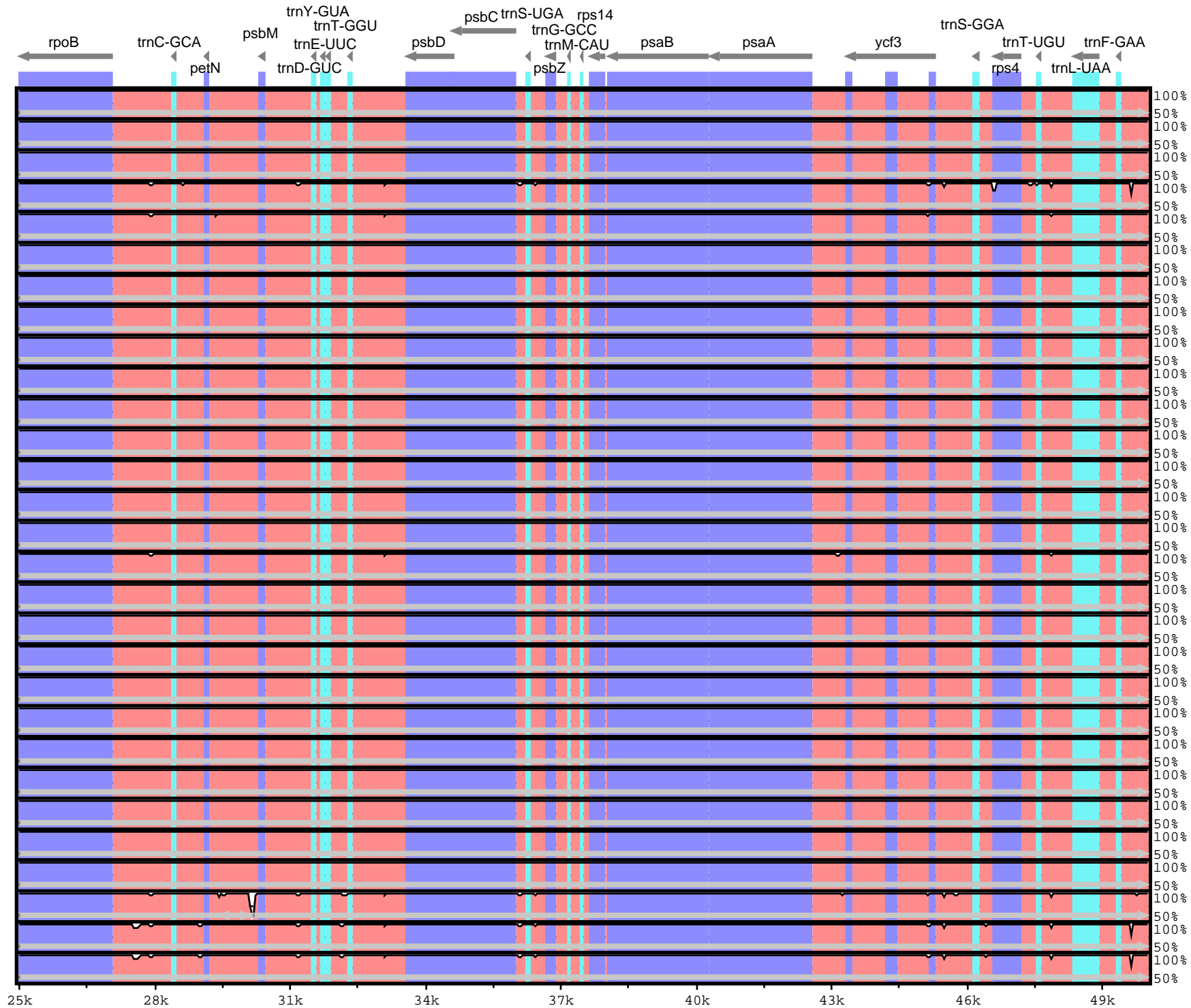

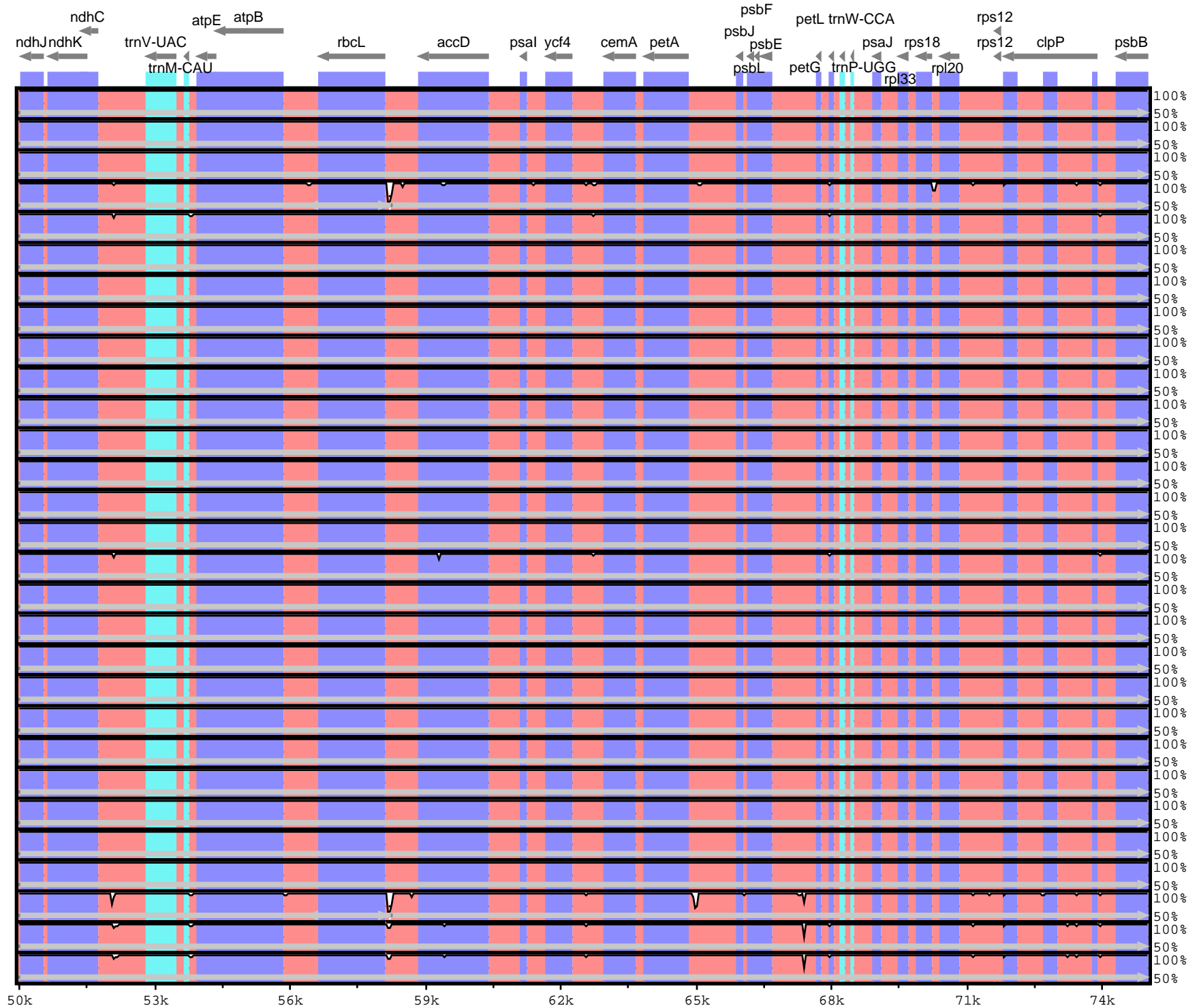

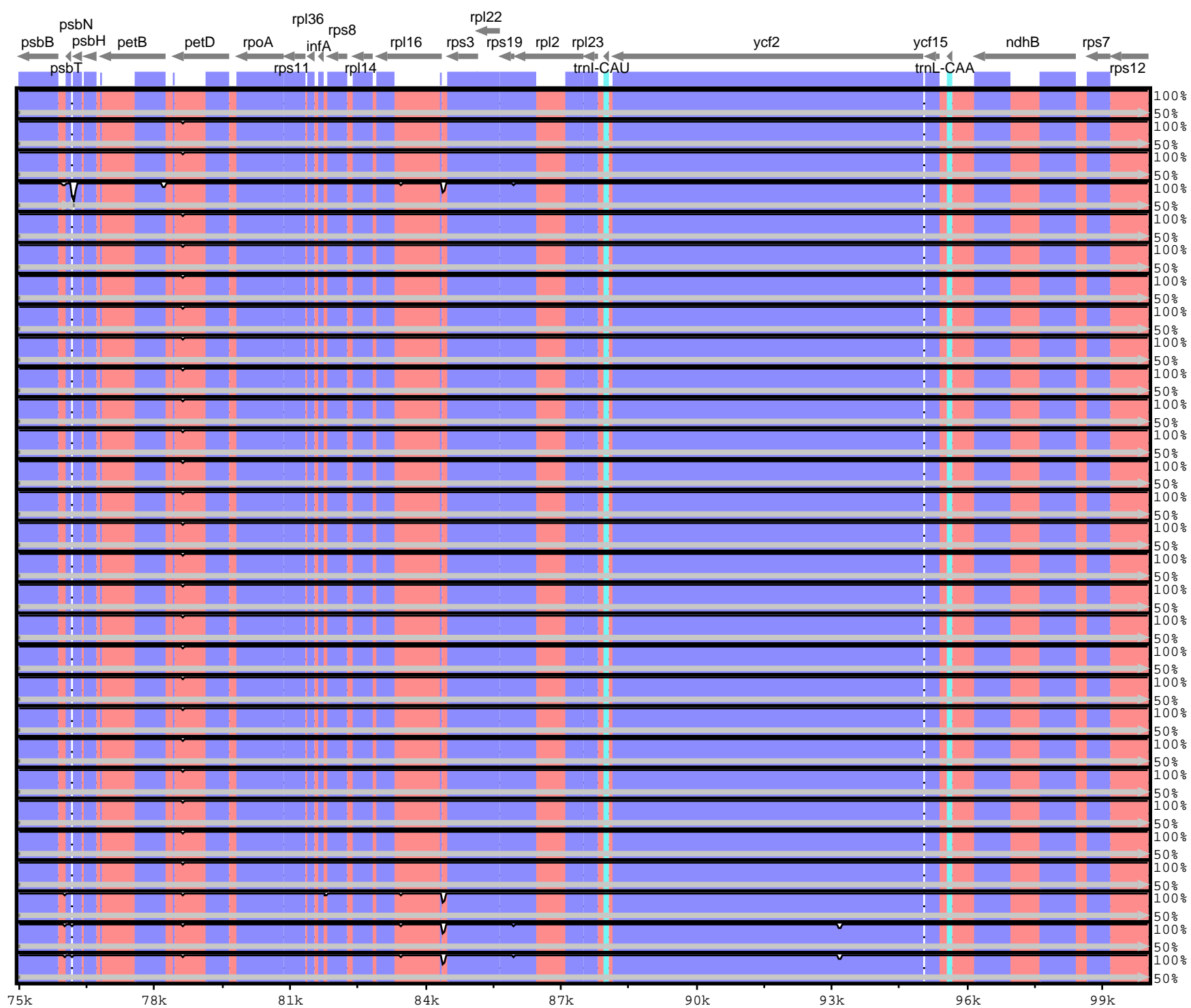

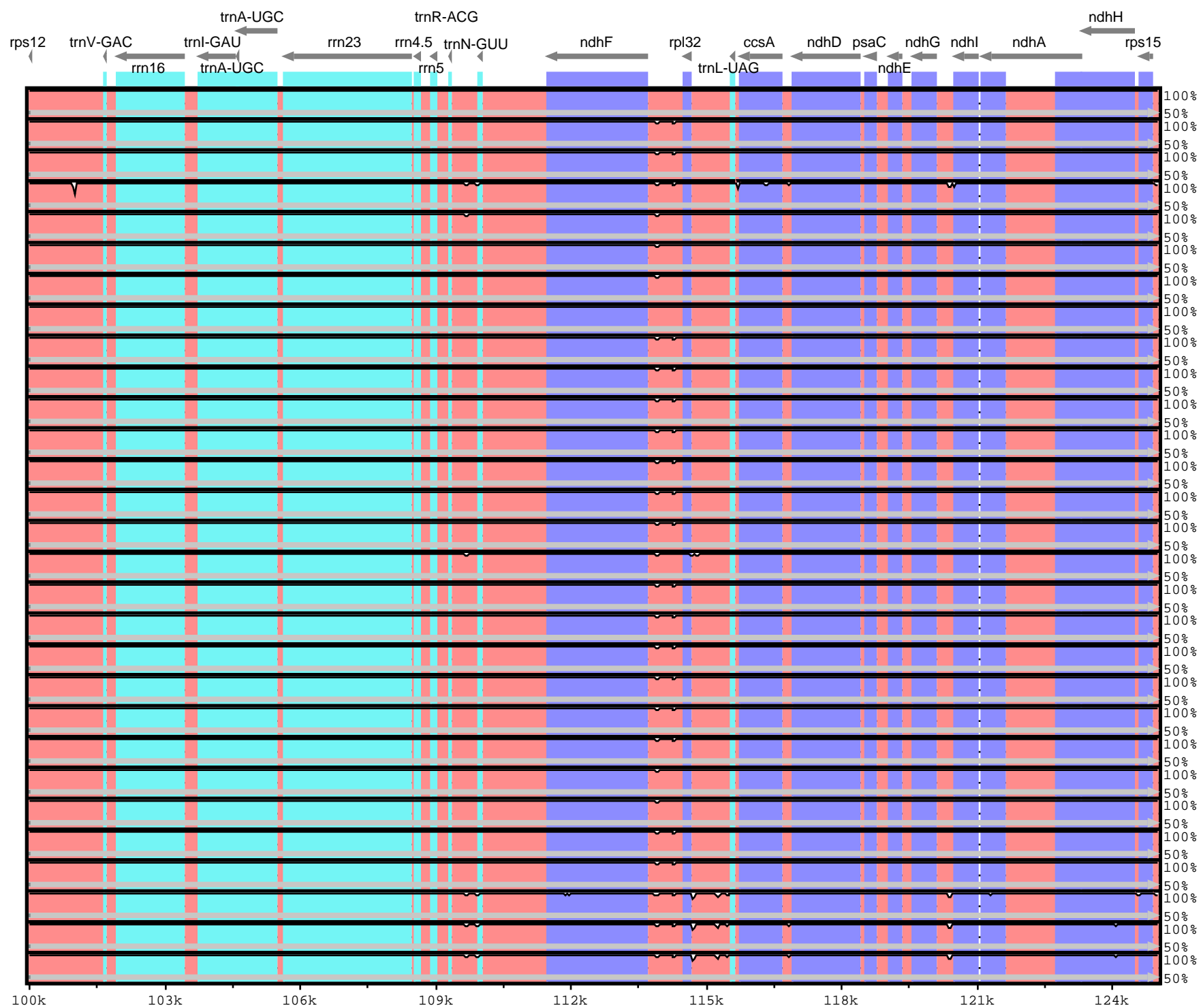

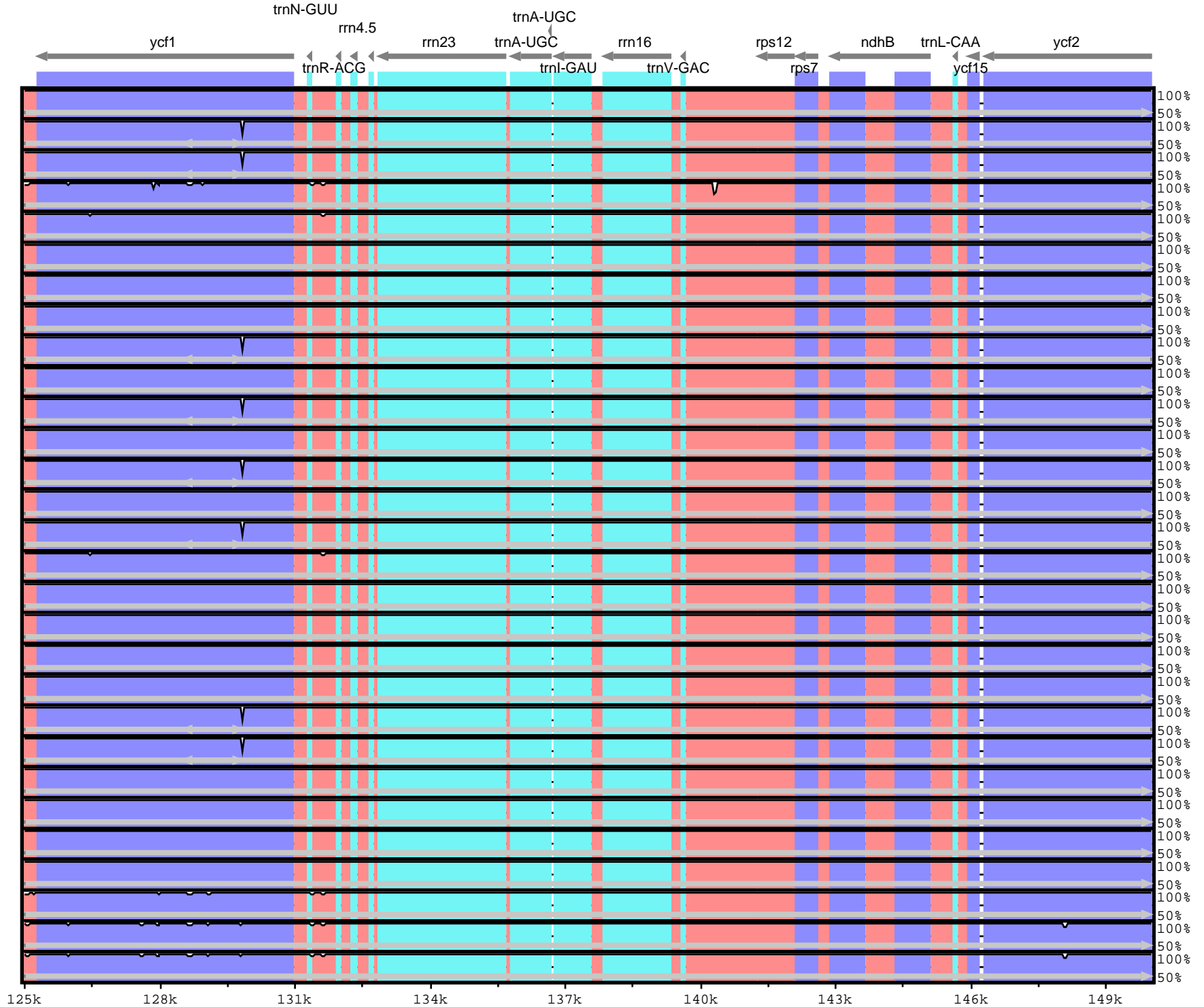

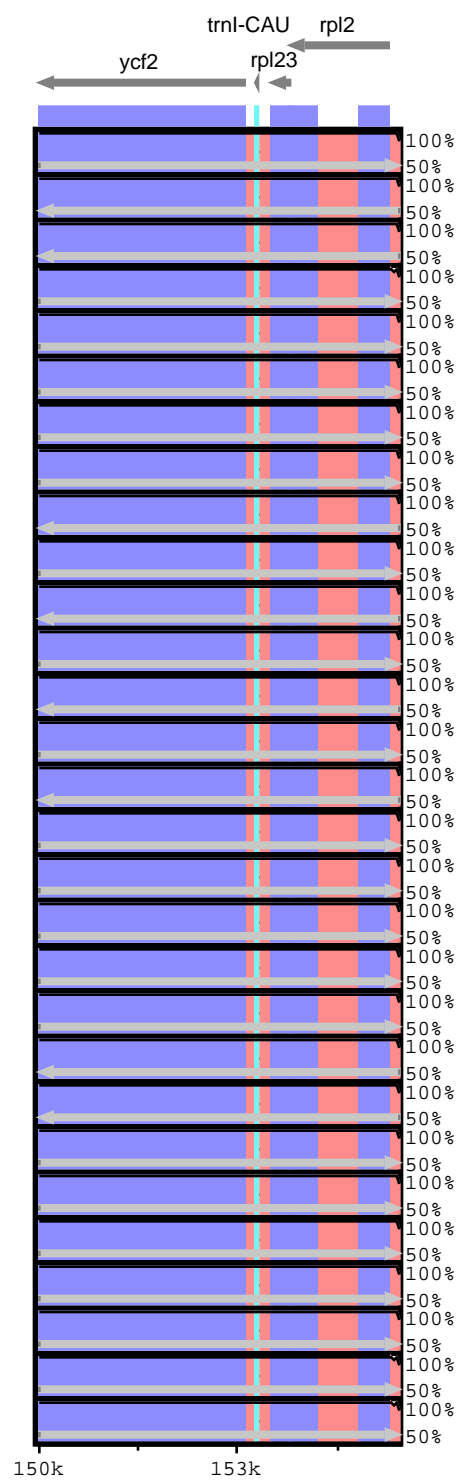

Supplement: Supplementary file 1 [file Image_1.pdf]

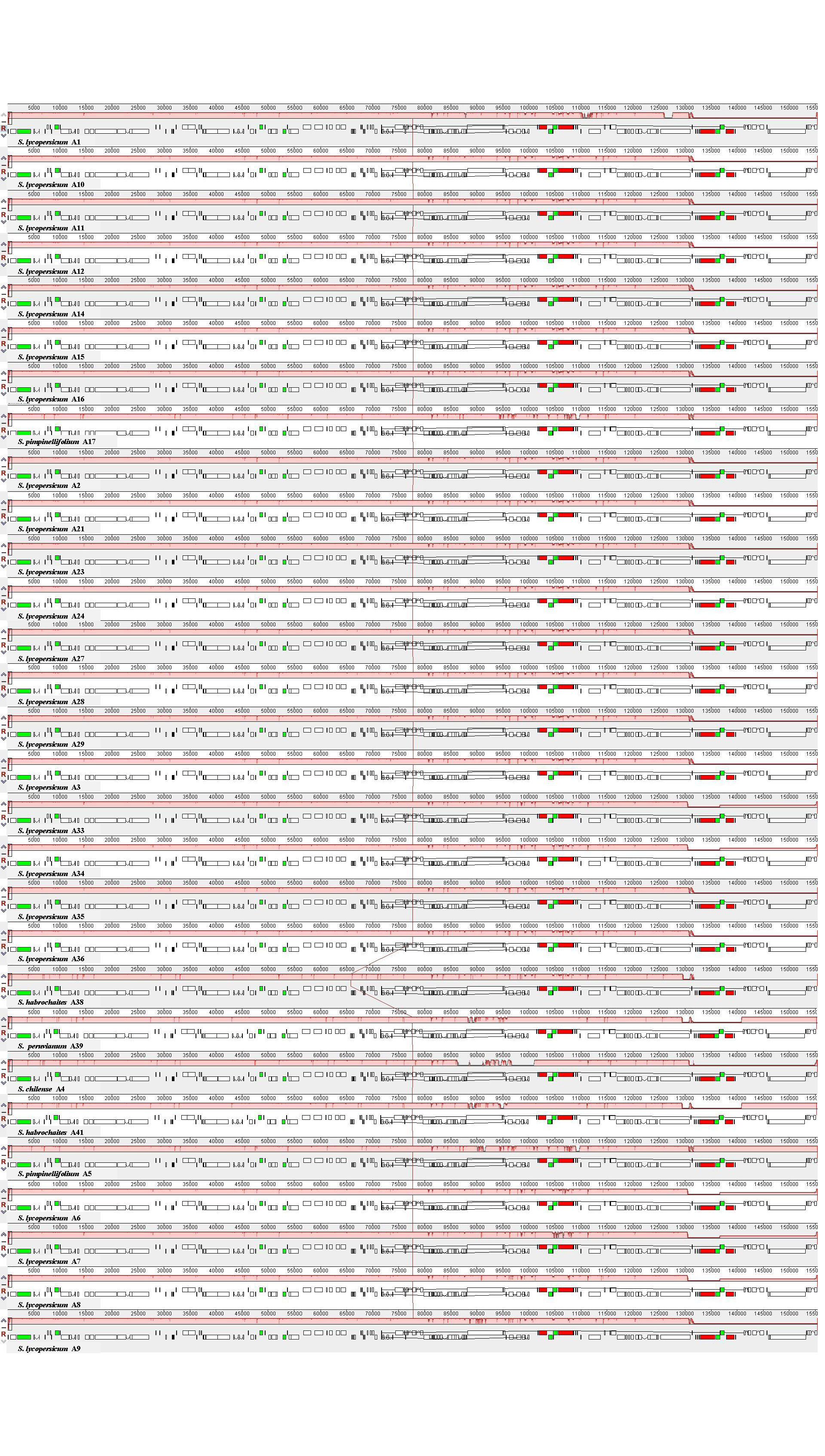

Supplement: Supplementary file 2 [file Image_2.tif]
