# Supplementary material for: Comparative analysis of chloroplast genomes of 29 tomato germplasms: genome structures, phylogenetic relationships, and adaptive evolution
Source: Front Plant Sci. 2023 May 9;14:1179009. doi: 10.3389/fpls.2023.1179009 (PMC10203424; doi:10.3389/fpls.2023.1179009)
Supplement: Supplementary file 3 [file Table_1.docx]

Supplementary Table 1. Summary of the chloroplast genomes of 29 tomato germplasm.

| **Sample name** | | **Genome Length**  **(bp)** | **LSC Length (bp)** | **IR Length (bp)** | **SSC Length (bp)** | **GC (%)** | **CDS** | **tRNA** | **rRNA** | **Total genes** |
| --- | --- | --- | --- | --- | --- | --- | --- | --- | --- | --- |
| A1 | 155435 | | 85849 | 25611 | 18364 | 37.86 | 80 | 29 | 4 | 113 |
| A2 | 155461 | | 85874 | 25612 | 18363 | 37.86 | 80 | 29 | 4 | 113 |
| A3 | 155461 | | 85874 | 25612 | 18363 | 37.86 | 80 | 29 | 4 | 113 |
| A4 | 155438 | | 85875 | 25594 | 18375 | 37.86 | 80 | 29 | 4 | 113 |
| A5 | 155450 | | 85866 | 25612 | 18360 | 37.87 | 80 | 29 | 4 | 113 |
| A6 | 155431 | | 85849 | 25611 | 18360 | 37.86 | 80 | 29 | 4 | 113 |
| A7 | 155431 | | 85849 | 25611 | 18360 | 37.86 | 80 | 29 | 4 | 113 |
| A8 | 155435 | | 85849 | 25611 | 18364 | 37.86 | 80 | 29 | 4 | 113 |
| A9 | 155461 | | 85874 | 25612 | 18363 | 37.86 | 80 | 29 | 4 | 113 |
| A10 | 155460 | | 85873 | 25612 | 18363 | 37.86 | 80 | 29 | 4 | 113 |
| A11 | 155461 | | 85874 | 25612 | 18363 | 37.86 | 80 | 29 | 4 | 113 |
| A12 | 155460 | | 85873 | 25612 | 18363 | 37.86 | 80 | 29 | 4 | 113 |
| A14 | 155461 | | 85874 | 25612 | 18363 | 37.86 | 80 | 29 | 4 | 113 |
| A15 | 155461 | | 85873 | 25612 | 18364 | 37.86 | 80 | 29 | 4 | 113 |
| A16 | 155461 | | 85874 | 25612 | 18363 | 37.86 | 80 | 29 | 4 | 113 |
| A17 | 155449 | | 85861 | 25612 | 18364 | 37.88 | 80 | 29 | 4 | 113 |
| A21 | 155460 | | 85873 | 25612 | 18363 | 37.86 | 80 | 29 | 4 | 113 |
| A23 | 155460 | | 85873 | 25612 | 18363 | 37.86 | 80 | 29 | 4 | 113 |
| A24 | 155460 | | 85873 | 25612 | 18363 | 37.86 | 80 | 29 | 4 | 113 |
| A27 | 155460 | | 85873 | 25612 | 18363 | 37.86 | 80 | 29 | 4 | 113 |
| A28 | 155461 | | 85874 | 25612 | 18363 | 37.86 | 80 | 29 | 4 | 113 |
| A29 | 155461 | | 85874 | 25612 | 18363 | 37.86 | 80 | 29 | 4 | 113 |
| A33 | 155431 | | 85849 | 25611 | 18360 | 37.86 | 80 | 29 | 4 | 113 |
| A34 | 155431 | | 85849 | 25611 | 18360 | 37.86 | 80 | 29 | 4 | 113 |
| A35 | 155460 | | 85873 | 25612 | 18363 | 37.86 | 80 | 29 | 4 | 113 |
| A36 | 155460 | | 85873 | 25612 | 18363 | 37.86 | 80 | 29 | 4 | 113 |
| A38 | 155422 | | 85845 | 25611 | 18355 | 37.84 | 80 | 29 | 4 | 113 |
| A39 | 155257 | | 85688 | 25607 | 18355 | 37.86 | 80 | 29 | 4 | 113 |
| A41 | 155257 | | 85688 | 25607 | 18355 | 37.86 | 80 | 29 | 4 | 113 |
