# Supplementary material for: Comparative analysis of chloroplast genomes of 29 tomato germplasms: genome structures, phylogenetic relationships, and adaptive evolution
Source: Front Plant Sci. 2023 May 9;14:1179009. doi: 10.3389/fpls.2023.1179009 (PMC10203424; doi:10.3389/fpls.2023.1179009)
Supplement: Supplementary file 4 [file Table_2.docx]

Supplementary Table 2. Genes in the chloroplast genome of 29 tomato germplasms.

| **Category** | **Gene group** | **Gene name** |
| --- | --- | --- |
| Miscellaneous group | Acetyl-CoA carboxylase | *accD* |
|  | Cytochrome c biogenesis | *ccsA* |
|  | Maturase | *matK* |
| Photosynthetic genes | Subunits of ATP synthase | *atpA, atpB, atpE, atpF, atpH, atpI* |
|  | Chloroplast envelope membrane protein | *cemA* |
|  | ATP-dependentprotease subumitP | *clpP* |
|  | Subunits of NADH dehydrogenase | *ndhA, ndhB, ndhC, ndhD, ndhE, ndhF, ndhG, ndhH, ndhI, ndhJ, ndhK* |
|  | Subumits of cytochrome | *petA, petB, petD, petG, petL, petN* |
|  | Subunits of photosystem I | *psaA, psaB, psaC, psaI, psaJ* |
|  | Subunits of photosystem II | *psbA, psbB, psbC, psbD, psbE, psbF, psbH, psbI, psbJ, psbK, psbL, psbM, psbN, psbT, psbZ* |
|  | The large subunit of Rubisco | *rbcL* |
| Transcription and translation-elated genes | Large subunit of ribosome | *rpl14, rpl16, rpl2, rpl20, rpl22, rpl23, rpl32, rpl33, rpl36* |
|  | Small subunit of the ribosome | *rps11, rps12, rps14, rps15, rps16, rps18, rps19, rps2, rps3, rps4, rps7, rps8* |
| Protein synthesis and DNAreplication | RNA polymerase | *rpoA, rpoB, rpoC1, rpoC2* |
| RNA genes | Ribosomal RNA genes | *rrn16, rrn23, rrn4.5, rrn5* |
|  | Transfer RNA genes | *trnA-UGC, trnC-GCA, trnD-GUC, trnE-UUC, trnF-GAA, trnG-GCC, trnH-GUG, trnI-CAU, trnI-GAU, trnK-UUU, trnL-CAA, trnL-UAA, trnL-UAG, trnM-CAU, trnN-GUU, trnP-UGG, trnQ-UUG, trnR-ACG, trnR-UCU, trnS-GCU, trnS-GGA, trnS-UGA, trnT-GGU, trnT-UGU, trnV-GAC, trnV-UAC, trnW-CCA, trnY-GUA, trnfM-CAU* |
| Pseudogene unknown function | Hypothetical chloroplast reading frames(ycf) | *ycf1, ycf15, ycf2, ycf3, ycf4* |
| Other gene | Translational initiation | *infA* |
