# Supplementary material for: Comparative analysis of chloroplast genomes of 29 tomato germplasms: genome structures, phylogenetic relationships, and adaptive evolution
Source: Front Plant Sci. 2023 May 9;14:1179009. doi: 10.3389/fpls.2023.1179009 (PMC10203424; doi:10.3389/fpls.2023.1179009)
Supplement: Supplementary file 6 [file Table_4.docx]

Supplementary Table 4. Distribution of distinct types of repeats in 29 tomato germplasm cp genomes.

| **Sample name** | **Simple sequence repeat** | | | | | **Tandem repeat** | **Dispersed repeat** | | | **Total** |
| --- | --- | --- | --- | --- | --- | --- | --- | --- | --- | --- |
|  | **A** | **T** | **TA** | **TTA** | **TAA** |  | **F** | **R** | **P** |  |
| A1 | 15 | 23 | 1 | - | - | 29 | 19 | - | 20 | **107** |
| A2 | 16 | 25 | 1 | - | - | 30 | 19 | - | 20 | **111** |
| A3 | 16 | 25 | 1 | - | - | 30 | 19 | - | 20 | **111** |
| A4 | 12 | 21 | 1 | 1 | - | 26 | 19 | 1 | 20 | **101** |
| A5 | 14 | 22 | 1 | - | - | 29 | 19 | 1 | 20 | **106** |
| A6 | 15 | 23 | 1 | - | - | 29 | 19 | - | 20 | **107** |
| A7 | 15 | 23 | 1 | - | - | 29 | 19 | - | 20 | **107** |
| A8 | 15 | 23 | 1 | - | - | 29 | 19 | - | 20 | **107** |
| A9 | 16 | 25 | 1 | - | - | 30 | 19 | - | 20 | **111** |
| A10 | 16 | 25 | 1 | - | - | 30 | 19 | - | 20 | **111** |
| A11 | 16 | 25 | 1 | - | - | 30 | 19 | - | 20 | **111** |
| A12 | 16 | 25 | 1 | - | - | 30 | 19 | - | 20 | **111** |
| A14 | 16 | 25 | 1 | - | - | 30 | 19 | - | 20 | **111** |
| A15 | 16 | 25 | 1 | - | - | 30 | 19 | - | 20 | **111** |
| A16 | 16 | 25 | 1 | - | - | 30 | 19 | - | 20 | **111** |
| A17 | 17 | 22 | 1 | - | - | 29 | 19 | - | 20 | **108** |
| A21 | 16 | 25 | 1 | - | - | 30 | 19 | - | 20 | **111** |
| A23 | 16 | 25 | 1 | - | - | 30 | 19 | - | 20 | **111** |
| A24 | 16 | 25 | 1 | - | - | 30 | 19 | - | 20 | **111** |
| A27 | 16 | 25 | 1 | - | - | 30 | 19 | - | 20 | **111** |
| A28 | 16 | 25 | 1 | - | - | 30 | 19 | - | 20 | **111** |
| A29 | 16 | 25 | 1 | - | - | 30 | 19 | - | 20 | **111** |
| A33 | 15 | 23 | 1 | - | - | 29 | 19 | - | 20 | **107** |
| A34 | 15 | 23 | 1 | - | - | 29 | 19 | - | 20 | **107** |
| A35 | 16 | 25 | 1 | - | - | 30 | 19 | - | 20 | **111** |
| A36 | 16 | 25 | 1 | - | - | 30 | 19 | - | 20 | **111** |
| A38 | 15 | 25 | 1 | - | 1 | 29 | 19 | - | 20 | **110** |
| A39 | 14 | 21 | 1 | - | - | 30 | 18 | 1 | 20 | **105** |
| A41 | 14 | 21 | 1 | - | - | 30 | 18 | 1 | 20 | **105** |
